# Supplementary material for: Factors Associated with SARS-CoV-2 Infection in Physician Trainees in New York City during the First COVID-19 Wave
Source: Int J Environ Res Public Health. 2021 May 15;18(10):5274. doi: 10.3390/ijerph18105274 (PMC8156350; doi:10.3390/ijerph18105274)
Supplement: Supplementary file 1 [file ijerph-18-05274-s001.zip › ijerph-1200960-supplementary.pdf]

Supplemental Table 1: Survey instrument to assess risk factors hypothesized to be associated with SARS-CoV-2 infection in trainees from February 1 through June 30, 2020

**Sociodemographic factors**
**What is your age?**
**Please select the sex you were assigned at birth:**<br>Female<br>Male<br>Other<br>Prefer not to answer
**Please select the racial or ethnic categories that best describe your heritage (you may select more than one):**<br>American Indian, Native American or Alaskan Native<br>Asian (East or Southeast Asian, South Asian / Indian)<br>Black / African American / African<br>Native Hawaiian or Other Pacific Islander<br>Caucasian / White<br>Other (specify)<br>Please specify the other racial and/or ethnic categories that best describe your heritage:
**Do you identify as Hispanic or Latinx?**<br>No<br>Yes
**Occupational factors**<br>Data provided by Graduate Medical Education office: training specialty, postgraduate year, training level (i.e., resident vs. fellowship status), primary hospital site
Work setting between February 1 and June 30, 2020
**Select the settings to which you were assigned from February 2020 through June 2020? (Select all that apply)**
Intensive care unit (ICU)<br>Medical/surgical (non-ICU)<br>Emergency department<br>Telemedicine<br>Ambulatory care/clinic<br>Other (specify)<br>Please specify any other setting to which you were assigned from February 1, 2020 through June 30, 2020:
High-risk occupational exposures between February 1 and June 30, 2020
**From February 2020 through June 2020, did you care for patients with COVID-19? (In-person only, do not consider telemedicine.)**<br>Yes

No

From February 2020 through June 2020, did you perform or attend an AEROSOL-GENERATING PROCEDURE performed on a patient under investigation (PUI) or with confirmed COVID-19? Please consider only the following procedures as aerosol-generating:

- **Cardiopulmonary resuscitation**
- **Endotracheal intubation and extubation**
- **Noninvasive positive pressure ventilation (e.g., BiPAP, CPAP)**
- **Manual ventilation**
- **Open suctioning of airway secretions**
- **Bronchoscopy**

Yes
No

Did you have CONTACT FOR MORE THAN 10 MINUTES with a patient under investigation (PUI) or with confirmed COVID-19 without wearing any of the following PPE during the period from February 1, 2020 through June 30, 2020?

- **Without wearing N95 respirator?**
- **Without wearing eye protection?**
- **Without wearing gown?**
- **Without wearing gloves?**

Yes, once
Yes, multiple times
No, never

Deployment factors between February 1 and June 30, 2020

From February 2020 through June 2020, were you assigned to patient care duties outside of your usual clinical site? E.g., your usual clinical sites are Mount Sinai Hospital and Bronx VA and you were assigned to work at Mount Sinai Brooklyn.

Yes
No

From February 2020 through June 2020, did your typical clinical activities significantly change? E.g., a pediatric resident caring for adult patients or an orthopedics resident working in an ICU

Yes
No

Select the type of change in your clinical activities from February 2020 through June 2020 (you may select more than one):

Patient population change (e.g., a pediatric resident treating adults)
Department change (e.g., from surgical resident working in the emergency department)
Setting change (e.g., from primarily outpatient to inpatient care)
More time dedicated to telemedicine

Other (specify)
**Community factors**
**Zip code of your primary residence during the period from February 2020 through June 2020**
**Have you had more than 10 minutes of contact OUTSIDE OF WORK with a person with confirmed or suspected SARS-CoV-2 infection from February 2020 through June 2020?**<br>Yes, with someone with confirmed infection<br>Yes, with someone with suspected infection<br>No
**Number of adults in your household (including yourself)**
**Number of children (< 18 years) in your household**
**What was your PRIMARY mode of transportation TO/FROM WORK from February 2020 through June 2020 (you may select more than one):**<br>Subway<br>Bus<br>Cab or rideshare (e.g., Uber, Lyft)<br>Personal vehicle<br>Bicycle<br>Walking<br>Commuter train<br>Ferry<br>Did not commute to/from work during this period<br>Other
**What was your PRIMARY mode of transportation in your frequent activities UNRELATED TO WORK (e.g., when going for groceries, or during your leisure time) from February 2020 through June 2020 (you may select more than one):**<br>Subway<br>Bus<br>Cab or rideshare (e.g., Uber, Lyft)<br>Personal vehicle<br>Bicycle<br>Walking<br>Commuter train<br>Ferry<br>Did not commute to/from work during this period<br>Other

Supplemental Table 2: Categorization of trainees who responded to the online survey between June 26 and August 31, 2020.

Training specialty	Hospital-based, primarily non-procedural (n = 213; 65%)	Surgery, OB-GYN or subspecialty (n =53; 16%)	High-risk, primarily procedural (n = 52; 16%)
**Adult Cardiothoracic Surgery**	0	2	0
**Advanced Minimally Invasive Surgery**	0	1	0
**Allergy and Immunology**	3	0	0
**Anatomic and Clinical Pathology**	2	0	0
**Anesthesiology**	0	0	22
**Breast Surgery**	0	1	0
**Cardiology CT/MR Imaging**	1	0	0
**Cardiothoracic Imaging**	1	0	0
**Cardiovascular Disease**	1	0	0
**Cardiovascular Disease (Investigator)**	2	0	0
**Cardiovascular EP**	1	0	0
**Child and Adolescent Psychiatry**	3	0	0
**Clinical Neurophysiology**	1	0	0
**Complex General Surgical Oncology**	0	1	0
**Critical Care Medicine**	0	0	2
**Dermatology**	6	0	0
**Dermatopharmacology**	1	0	0
**Diagnostic Radiology**	11	0	0
**Emergency Medicine**	0	0	21
**Endocrinology**	1	0	0
**Endocrinology, Diabetes and Metabolism**	5	0	0
**Family Medicine**	11	0	0
**Female Pelvic Medicine and Reconstructive Surgery**	0	1	0
**Gastroenterology**	5	0	0
**General Practice Dentistry**	0	3	0
**Geriatric Medicine**	7	0	0
**Head and Neck Surgery**	0	4	0
**Headache Medicine**	1	0	0
**Hematology and Medical Oncology**	7	0	0
**Hospice and Palliative Medicine**	5	0	0
**Inflammatory Bowel Diseases**	1	0	0
**Infectious Disease**	6	0	0
**Internal Medicine**	58	0	0
**Liaison Psychiatry**	1	0	0

**Medicine**	1	0	0
**Movement Disorders**	1	0	0
**Multiple Sclerosis**	1	0	0
**Neonatal Perinatal Medicine**	0	2	0
**Nephrology**	2	0	0
**Neurocritical Care**	0	0	1
**Neurology**	9	0	0
**Neuromuscular Medicine**	1	0	0
**Neuroradiology**	1	0	0
**Neurosurgery**	0	1	0
**Obstetrics and Gynecology**	0	8	0
**Occupational Medicine**	1	0	0
**Ophthalmology**	0	1	0
**Oral and Maxillofacial**	0	1	0
**Orthopedic Surgery**	0	7	0
**Oral and Maxillofacial Surgery**	0	1	0
**Pediatric Gastroenterology**	1	0	0
**Pediatric Inflammatory Bowel Diseases**	1	0	0
**Pediatrics**	17	0	0
**Pediatrics/Medical Genetics**	1	0	0
**Pediatrics/Psychiatry/Child and Adolescent Psychiatry**	3	0	0
**Physical Medicine and Rehabilitation**	2	0	0
**Podiatric Medicine and Surgery**	0	1	0
**Preventive Medicine**	2	0	0
**Procedural Dermatology**	0	1	0
**Psychiatry**	24	0	0
**Pulmonary Disease and Critical Care Medicine**	0	0	5
**Radiation Oncology**	2	0	0
**Regional Anesthesiology and Acute Pain Medicine**	0	0	1
**Rheumatology**	2	0	0
**Shoulder Orthopedics**	0	1	0
**Surgery**	0	12	0
**Thoracic Surgery Integrated**	0	2	0
**Urology**	0	2	0

Supplemental Table 3: Self-reported SARS-CoV-2 test results

Variable	Negative SARS-CoV-2 test (n = 262)	Positive SARS-CoV-2 test (n = 66)
RT- PCR (nasal or oral swab) only, no. (%)	12 (5)	2 (3)
IgG antibodies only, no. (%)	158 (60)	47 (71)
RT- PCR and IgG antibodies, no. (%)	92 (35)	17 (26)

Abbreviations: RT-PCR, reverse transcriptase-polymerase chain reaction.

Supplemental Table 4: Comparison of main characteristics between study participants (n=328), the randomly selected sample (n=62) and all initially eligible trainees (n=2543)

Variable	Study participants (n = 328)	Randomly selected sample (n = 62)		All eligible trainees (n = 2543)	
	n (%)	n (%)	*P*-value[a]	n (%)	*P*-value[b]
**Primary hospital site, n (%)**			0.74		0.01
Beth Israel Medical Center	28 (9)	2 (3)		232 (9)	
Elmhurst Hospital Center	15 (5)	3 (5)		157 (6)	
Institute for Family Health	6 (2)	1 (2)		23 (1)	
Mount Sinai Medical Center	211 (64)	40 (65)		1403 (55)	
New York Eye and Ear Infirmary	0 (0)	0 (0)		27 (1)	
North Central Bronx	1 (0)	0 (0)		32 (1)	
Queens Hospital Center	7 (2)	1 (2)		87 (3)	
South Nassau Communities Hospital	4 (1)	0 (0)		71 (3)	
St. Luke's Roosevelt Hospital	56 (17)	15 (24)		511 (20)	
**PGY level, n (%)**			0.35		0.64
1	73 (22)	16 (26)		605 (24)	
2	62 (19)	7 (11)		512 (20)	
≥ 3	193 (59)	39 (63)		1426 (56)	
**Residency or fellowship, n (%)**			0.44		0.27
Fellowship	85 (26)	19 (31)		589 (23)	
Residency	243 (74)	43 (69)		1954 (77)	
**Self-reported SARS-CoV-2 test result**			0.22		
Negative	262 (80)	54 (87)			
Positive	66 (20)	8 (13)			
**Race**			0.28		
White	208 (63)	40 (65)			
Asian	82 (25)	19 (31)			
Black	26 (8)	1 (2)			
Other	12 (4)	2 (3)			
**Hispanic/Latinx**			0.48		
No	294 (90)	58 (94)			
Yes	34 (10)	4 (6)			
**Change in usual patient population**			0.65		
No	296 (90)	55 (89)			
Yes	32 (10)	7 (11)			
**Medical/surgical unit**			>0.99		
No	106 (32)	20 (32)			
Yes	222 (68)	42 (68)			
**Ambulatory clinic**			0.23		
No	227 (69)	48 (77)			
Yes	101 (31)	14 (23)			

**Contact >10 mins *without N95* with a PUI or confirmed COVID-19 case**			0.61
Never	221 (67)	38 (61)	
Once	55 (17)	12 (19)	
More than once	52 (16)	12 (19)	
**Training specialty**			0.47
Hospital based, primarily non-procedural	221 (67)	38 (61)	
High-risk procedural	53 (16)	14 (23)	
Surgery/subspecialty	54 (17)	10 (16)	
**Number of children in household**			0.85
0	275 (84)	53 (86)	
≥ 1	53 (16)	9 (14)	
**Contact with individual with confirmed or suspected COVID-19 outside of work**			0.41
Yes	73 (22)	17 (27)	
No	255 (78)	45 (73)	
**Primary mode of transportation to locations other than work: *subway or bus***			0.86
No	269 (82)	52 (84)	
Yes	59 (18)	10 (16)	
**Primary mode of transportation to locations other than work*: private car, bike, walk***			0.22
No	19 (6)	1 (2)	
Yes	309 (94)	61 (98)	
**Primary residence (zip code)**			0.98
Manhattan	264 (81)	52 (84)	
Queens	30 (9)	5 (8)	
Brooklyn	12 (4)	2 (3)	
Bronx	5 (2)	1 (2)	
Other (outside of NYC)	17 (5)	2 (3)	

Abbreviations: PGY, post-graduate year.

[a]Statistical comparison between study sample and randomly selected sample to increase study participation

[b]Statistical comparison between study sample and all trainees

Supplemental Table 5: Sensitivity analysis

Variable	Final adjusted model		Excluding trainees without IgG antibody results		Adjustment for date of SARS-CoV-2 test	
	(n = 328)		(n = 314)		(n = 186)	
	OR	95% CI	OR	95% CI	OR	95% CI
**Race**						
White (ref)	1.00	-	1.00	-	1.00	-
Asian	0.53	0.24, 1.15	0.56	0.26, 1.23	0.52	0.20, 1.33
Black	1.42	0.50, 4.01	1.17	0.40, 3.39	0.61	0.16, 2.36
Other	0.64	0.14, 2.92	0.51	0.10, 2.57	0.84	0.14, 4.93
**Hispanic/Latinx**						
No (ref)	1.00	-	1.00	-	1.00	-
Yes	1.98	0.72,5.46	1.96	0.70, 5.50	2.11	0.68, 6.57
**Change in usual patient population**						
No (ref)	1.00	-	1.00	-	1.00	-
Yes	0.16	0.03, 0.73	0.16	0.04, 0.73	0.22	0.05, 1.04
**Medical/surgical unit**						
No (ref)	1.00	-	1.00	-	1.00	-
Yes	2.51	1.18, 5.34	2.40	1.12, 5.13	1.54	0.63, 3.80
**Ambulatory clinic**						
No (ref)	1.00	-	1.00	-	1.00	-
Yes	0.61	0.29, 1.30	0.57	0.27, 1.23	0.55	0.21, 1.42
**Contact >10 mins *without N95* with a PUI or confirmed COVID-19 case**						
Never (ref)	1.00	-	1.00	-	1.00	-
Once	1.24	0.55, 2.75	1.19	0.54, 2.66	1.15	0.44, 2.98
More than once	1.59	0.74, 3.43	1.43	0.65, 3.18	1.37	0.53, 3.52
**Training specialty**						
Primarily non-procedural	1.00	-	1.00	-	1.00	-
High-risk procedural	2.93	1.24, 6.92	2.78	1.18, 6.58	2.56	0.88, 7.42
Surgery/subspecialty	1.51	0.65, 3.50	1.33	0.56, 3.13	1.15	0.44, 2.99
**Number of children in household**						
0	1.00	-	1.00	-	1.00	-
≥ 1	0.59	0.23, 1.48	0.60	0.24, 1.53	0.57	0.18, 1.86
**Contact with individual with confirmed or**						

**suspected COVID-19 outside of work**						
No	1.00	-	1.00	-	1.00	-
Yes	1.58	0.78, 3.17	1.51	0.74, 3.07	1.71	0.69, 4.26
**Primary mode of transportation to locations other than work:** ***subway or bus***						
No	1.00	-	1.00	-	1.00	-
Yes	1.85	0.85, 3.99	1.84	0.84, 4.02	1.74	0.64, 4.77
**Primary mode of transportation to locations other than work*****: private car, bike, walk***						
No	1.00	-	1.00	-	1.00	-
Yes	0.42	0.14, 1.27	0.39	0.13, 1.20	0.36	0.09, 1.47
**Primary residence (zip code)**						
Manhattan	1.00	-	1.00	-	1.00	-
Queens	0.34	0.10, 1.20	0.40	0.11, 1.40	0.34	0.06, 1.90
Brooklyn	0.30	0.06, 1.62	0.32	0.06, 1.77	0.37	0.06, 2.09
Bronx	0.48	0.08, 3.08	0.57	0.09, 3.76	-	-
Other (outside of NYC)	1.51	0.44, 5.20	1.65	0.47, 5.76	1.91	0.36, 10.21
**Date of test**	-	-	-	-	1.33	0.94, 1.86

Abbreviations: OR, odds ratio; CI, confidence interval; ref, reference.
